# Biosynthesis, Antimicrobial and Cytotoxic Effect of Silver Nanoparticles Using a Novel *Nocardiopsis* sp. MBRC-1

**DOI:** 10.1155/2013/287638

**Published:** 2013-07-11

**Authors:** Panchanathan Manivasagan, Jayachandran Venkatesan, Kalimuthu Senthilkumar, Kannan Sivakumar, Se-Kwon Kim

**Affiliations:** ^1^Marine Biotechnology Laboratory, Department of Chemistry and Marine Bioprocess Research Center, Pukyong National University, Busan 608-737, Republic of Korea; ^2^Department of Chemistry and Marine Bioprocess Research Center, Pukyong National University, Busan 608-737, Republic of Korea; ^3^Centre of Advanced Study in Marine Biology, Faculty of Marine Sciences, Annamalai University, Parangipettai, Tamil Nadu 608 502, India

## Abstract

The biosynthesis of nanoparticles has been proposed as a cost effective environmental friendly alternative to chemical and physical methods. Microbial synthesis of nanoparticles is under exploration due to wide biomedical applications, research interest in nanotechnology and microbial biotechnology. In the present study, an ecofriendly process for the synthesis of nanoparticles using a novel *Nocardiopsis* sp. MBRC-1 has been attempted. We used culture supernatant of *Nocardiopsis* sp. MBRC-1 for the simple and cost effective green synthesis of silver nanoparticles. The reduction of silver ions occurred when silver nitrate solution was treated with the *Nocardiopsis* sp. MBRC-1 culture supernatant at room temperature. The nanoparticles were characterized by UV-visible, TEM, FE-SEM, EDX, FTIR, and XRD spectroscopy. The nanoparticles exhibited an absorption peak around 420 nm, a characteristic surface plasmon resonance band of silver nanoparticles. They were spherical in shape with an average particle size of 45 ± 0.15 nm. The EDX analysis showed the presence of elemental silver signal in the synthesized nanoparticles. The FTIR analysis revealed that the protein component in the form of enzyme nitrate reductase produced by the isolate in the culture supernatant may be responsible for reduction and as capping agents. The XRD spectrum showed the characteristic Bragg peaks of 1 2 3, 2 0 4, 0 4 3, 1 4 4, and 3 1 1 facets of the face centered cubic silver nanoparticles and confirms that these nanoparticles are crystalline in nature. The prepared silver nanoparticles exhibited strong antimicrobial activity against bacteria and fungi. Cytotoxicity of biosynthesized AgNPs against in vitro human cervical cancer cell line (HeLa) showed a dose-response activity. IC_50_ value was found to be 200 **μ**g/mL of AgNPs against HeLa cancer cells. Further studies are needed to elucidate the toxicity and the mechanism involved with antimicrobial and anticancer activity of the synthesized AgNPs as nanomedicine.

## 1. Introduction

Nanotechnology is emerging as a rapidly growing field with its application in science and technology [1]. Noble metal nanoparticles such as gold, silver, and platinum are widely applied in medicinal applications. Marine actinobacteria are high Guanine+Cytosine content Gram-positive bacteria with an unparalleled ability to produce diverse secondary metabolites, such as antibiotics, immunosuppressors, and many other biologically active compounds [2]. Exploitation of marine actinobacteria in nanotechnology has recently received considerable attention [3, 4]. Nanotechnology holds promising application in biosensing, drug delivery, and cancer therapy [5–7]. The expensive and extensive use of toxic solvents and hazardous reducing agents in chemical procedures to synthesize nanoparticles has augmented the necessity in view of ecofriendly and green chemistry approach. Hence, a well established nontoxic and ecofriendly potent methodology for the synthesis of nanoparticles has mounted to a level of supreme importance [8–11]. An alternative approach for the synthesis of metal nanoparticles is to apply biomaterials such as plants, microorganisms encompassing groups such as bacteria, fungi, and actinobacteria as nanofactories [12–14]. Emerging multidrug resistant (MDR) bacteria has raised a demand for the urgent need to identify novel antimicrobial agents. It was reported that silver had been used as antimicrobial agents since ancient times [3]. With the advancements in nanotechnology, AgNPs have found its significant applications as antimicrobial agents, in fields of microelectronics, catalysis, and biomolecular detection [15–17]. Although the antibacterial activity of AgNPs has been proved in the recent years, the actual mechanism of action is not yet clear. They may inactivate microorganisms by interacting with their enzymes, proteins, or DNA to inhibit cell proliferation [18]. It is also evident that the increased antimicrobial activity of AgNPs may be attributed to its special characteristics of small size and high surface area to volume ratio [19]. The advantage of adapting biosynthesis of AgNPs is the simplicity of extracellular synthesis and downstream processing [20, 21].

Nanoparticles have a wide range of applications, as in combating microbes [22], biolabelling [23], and in the treatment of cancer [24]. The antibacterial activity of silver species is known since ancient times [25] and it has been demonstrated that, at low concentrations, silver is nontoxic to human cells [26]. It has also been reported that Ag^+^ ions uncouple the respiratory chain from oxidative phosphorylation or collapse the proton-motive force across the cytoplasmic membrane [27]. The interaction of Ag^+^ with bacteria is directly related to the size and shape of the nanoparticles [26, 28]. 

Sastry et al. [29] reported on the biosynthesis of metal nanoparticles using the mycelial extract of fungi and actinobacteria [29]. In addition, the time required for completion of the reaction using both bacteria and fungi ranges between approximately 24 hrs and 120 hrs, whereas maximum synthesis of AgNPs can be achieved after 24 hrs of incubation. Moreover, metal accumulation is dependent on the growth phase of the cells [30]. Sadhasivam et al. [3] reported on the extracellular biosynthesis of NPs by *Streptomyces hygroscopicus* and antimicrobial activity against medically important pathogenic micro-organisms [3]. Sivalingam et al. [31] reported on the biosynthesis of bactericidal silver nanoparticles (AgNPs) using a novel *Streptomyces* sp. BDUKAS10, an isolated mangrove sediment [31]. Though the mechanism of silver resistance offered by bacteria using the silver binding protein is well documented, their extraction and purification need to be elucidated further for large-scale production. However, only a few studies have examined the components of marine actinobacteria that mediated the reduction of silver ions into AgNPs. In this study, we examined and characterized the extracellular biosynthesis of AgNPs using a novel *Nocardiopsis* sp. MBRC-1, which is a very important micro-organism to the production of several antibiotics and enzymes of commercial value. To the best of our knowledge, this marine actinobacterium (*Nocardiopsis* sp. MBRC-1) has never been used for nanoparticles biosynthesis.

## 2. Materials and Methods

### 2.1. Chemicals

All analytical reagents and media components were purchased from Sigma-Aldrich (St. Louis, USA).

### 2.2. Microbial Synthesis of AgNPs

The *Nocardiopsis* sp. MBRC-1 strain was isolated from the marine sediment samples from the Busan coast (Lat 35°09′ N; Long 129°07′ E), South Korea. Their partial 16S rRNA gene sequences were deposited in GenBank under the accession number KC179785. For the synthesis of silver nanoparticles, the active *Nocardiopsis* sp. MBRC-1 culture was freshly inoculated on sterile starch casein medium and the flasks were incubated at 25–28°C and 180 rpm for 96 hrs (pH 7.0). After the incubation period was complete, the culture was centrifuged at 5000 rpm for 30 min and the supernatant was used for the biosynthesis of AgNPs. Deionized water was used as a solvent in the synthesis of AgNPs. The collected supernatant (pH 7.0) was added separately to the reaction vessel containing silver nitrate at a concentration of 10^−3 ^M (1% (v/v)) and incubated on an orbital shaker (dark condition) for 96 hrs at 30°C. The reaction was carried out in the dark after the addition of the AgNO_3_, and color change appeared transparent. It confirmed the synthesis of AgNPs. The formation of the AgNPs was monitored by UV-vis spectroscopy using Shimadzu (Model No-UV 1800) double beam UV-vis spectrophotometer [3]. All the experiments were carried out in triplicate and average values have been reported.

### 2.3. Characterization of AgNPs

The synthesized AgNPs were freeze dried, powdered, and used for XRD analysis. The spectra were evaluated using an X-ray diffractometer (PHILIPS X'Pert-MPD diffractometer, The Netherlands) and Cu-K**α**radiation 1.5405 Å over an angular range of 5 to 80°, a step size of 0.02, a scan speed of 4° m^−1^ at a 40 kV voltage, and a 30 mA current. The dried powder was diluted with potassium bromide in the ratio of 1 : 100 and recorded the Fourier transform infrared spectroscopy (FTIR) (Perkin Elmer Inc., USA) and spectrum GX spectrometry within the range of 400 to 4000 cm^−1^. Synthesized AgNPs were mounted on specimen stubs with double-sided adhesive tape coated with platinum in a sputter coater and examined under field emission scanning electron microscopy (FE-SEM) (JSM-6700, JEOL, Japan). For transmission electron microscopy (TEM) imaging, a drop of aqueous solution containing the AgNPs was placed on carbon coated copper grids and dried under an infrared lamp (JEM 1010 JEOL, Japan) (AC voltage −80.0 kV). In addition, the presence of silver metals in the sample was analyzed by energy dispersive X-ray analysis (EDX) combined with FE-SEM. Finally, the size distribution of the nanoparticles was evaluated using dynamic light scattering measurements conducted with a Malvern Zetasizer ZS compact scattering spectrometer (Malvern Instruments Ltd., Malvern, UK).

### 2.4. Particle-Size Distribution of AgNPs

Particle-size distribution analysis was carried out after treatment of a 1 mM solution of AgNO_3_ with the culture supernatant of *Nocardiopsis* sp. MBRC-1 at room temperature for 98 hrs. The organism was grown in starch casein broth under incubation at 30°C for 98 hrs. After the incubation period, the culture was centrifuged at 10,000 rpm and the supernatant was used to reduce the AgNO_3_ solution. For the DLS measurements, the supernatant thus obtained was a clear brown homogenous suspension of AgNPs diluted 10-fold for all experiments involving measurement of DLS. The solutions were then filtered through syringe membrane filters with pores less than 0.4 *μ*m, then centrifuged at 5000 rpm for 30 min.

### 2.5. Antimicrobial Activity of the AgNPs

The antimicrobial activity of the microbiologically synthesized AgNPs against pathogenic organisms such as bacteria (*Escherichia coli*, *Bacillus subtilis*, *Enterococcus hirae*, *Pseudomonas aeruginosa*, *Shigella flexneri* and *Staphylococcus aureus*) and fungi (*Aspergillus niger, A. brasiliensis, A. fumigates *and* Candida albicans*) was measured using the well-diffusion method [26]. Pure cultures of bacteria and fungi were grown in Mueller-Hinton broth (Sigma, USA) for bacteria and Sabouraud-broth for fungi at 35°C and 30°C, respectively, on a rotary shaker at 180 rpm. Wells that were 6 mm in diameter were made on the Mueller-Hinton agar and Sabouraud agar plates using a gel puncture and each well was inoculated with individual cultures. The AgNPs in various concentrations (10, 20, 30, 40, and 50 *μ*g/mL) were loaded in each well. The positive and negative controls were also maintained, and the plates (triplicates) were incubated at 35°C and 30°C for 24 and 48 hrs. Simultaneously, the synergistic effects of different commercial antibiotics (Amoxicillin and Nystatin, Sigma, USA) with AgNPs against multidrug resistant pathogens were also checked in well diffusion method. After incubation, the susceptibility pattern of the test organisms was determined by measuring the diameter of the zone of inhibition for well diffusion method.

### 2.6. Determination of Minimum Inhibitory Concentration

The synthesized silver nanoparticles were tested (triplicates) for minimum inhibitory concentration by microtiter broth dilution method [32]. Muller-Hinton broth was used as diluents for bacterial strains and Sabouraud broth for fungal species. About 10^6^ CFU/mL cells were inoculated, and the final volume in each microtiter plate well was 0.1 mL. After incubation for 24 h, at 35°C for bacterial strains and 30°C for fungal strains, the microtiter plates were read at 450 nm using TRIAD multimode reader prior to and after incubation to determine the minimum inhibitory concentration (MIC) values. The MIC is defined as the lowest concentration of compound, which inhibited 90% of the growth when compared with that of the growth control.

### 2.7. Cell Culture

Human cervical cancer cell line (HeLa) was cultured in Dulbecco's Modified Eagle Medium (DMEM). Culture media were supplemented with 10% fetal bovine serum (FBS) and 1% antibiotic and antimycotic (Penicillin-Streptomycin cocktail) solution. The cells were grown in a humidified atmosphere containing 5% CO_2_ at 37°C and subcultured by detaching with trypsin-EDTA solution at about 70–80% confluent.

### 2.8. Cytotoxic Activity

Cell viability was evaluated by the MTT colorimetric technique. Human HeLa cancer cell lines (5000 cells/well) were seeded in 96 well tissue culture plates. Stock solutions of nanoparticles (5 mg/mL) were prepared in sterile distilled water and diluted to the required concentrations (50, 100, 150, 200, and 250 *μ*g/mL) using the cell culture medium. Appropriate concentrations of AgNPs stock solution were added to the cultures to obtain respective concentration of AgNPs and incubated for 24 hrs at 37°C. Nontreated cells were used as control. After 24 hrs, cells were washed with PBS and then 100 *μ*L of the yellow tetrazolium MTT solution (3-(4,5-dimethylthiazolyl-2)-2,5-diphenyltetrazolium bromide) without phenol red (0.5 mg/mL in phosphate buffer solution) was added to each well. The plates were incubated for 3-4 hrs at 37°C, for reduction of MTT by metabolically active cells, in part by the action of dehydrogenase enzymes, to generate reducing equivalents such as NADH and NADPH. For solubilization of the MTT crystals, 100 *μ*L of DMSO was added to the wells. The plates were placed on a shaker for 15 min to complete solubilization of crystals, and then the optical density of each well was determined. The quantity of formazan product as measured by the amount of 545 nm absorbance is directly proportional to the number of living cells in culture. Each experiment was done in triplicate. The relative cell viability (%) related to control wells containing cell culture medium without nanoparticles as a vehicle was calculated as follows: Percentage of cell viability (%) = Sample absorbance/control absorbance × 100.

### 2.9. Cytomorphological Changes in HeLa Cells by AgNPs

HeLa cells (1 × 10^5^ cells/well) were seeded in a 6 well plate for 24 hrs. After 24 hrs, they were treated with 100 and 200 *μ*g/mL of synthesized AgNPs and incubated for 24 hrs at 37°C in 5% CO_2_ atmosphere. After the incubation, the cells were washed twice with PBS, and morphological changes in the cells were visualized and photographed under phase contrast microscope (CTR 6000; Leica, Wetzlar, Germany).

### 2.10. Statistical Analysis

The grouped data were statistically evaluated using ANOVA with SPSS/14 software. Values are presented as the mean ± SD of the three replicates of each experiment.

## 3. Results and Discussion

### 3.1. Isolation and Identification of Marine Actinobacteria

**Figure 1:**
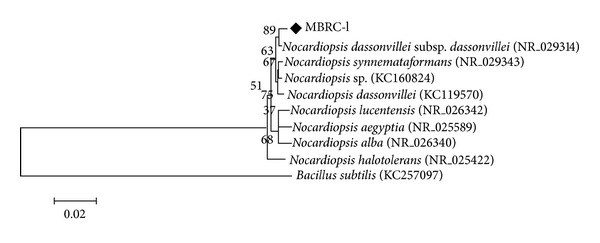
Phylogenetic tree of the 16S rDNA sequence of strain *Nocardiopsis* sp. MBRC-1 and related strains.

A marine actinobacterium MBRC-1 strain was isolated from the marine sediment samples from the Busan coast, South Korea, and was used for the synthesis of silver nanoparticles. The marine actinobacterium MBRC-1 shows that the presence of *meso-*diaminopimelic acid as the amino acid in the cell wall and arabinose and galactose as whole cell sugars and the absence of characteristic glycine in their cell credibly categorized the cell wall of this strain belonged to the cell wall type-IV [33]. This isolate was identified as *Nocardiopsis* sp. MBRC-1 based on the morphological, physiological, and biochemical characteristics, and it was confirmed by the 16S rDNA sequencing (Figure 1). The sequence was submitted to GenBank in NCBI (http://www.ncbi.nlm.nih.gov/nuccore/443501390/) with the accession number KC179785. 

### 3.2. UV-Vis Analysis of AgNPs

**Figure 2:**
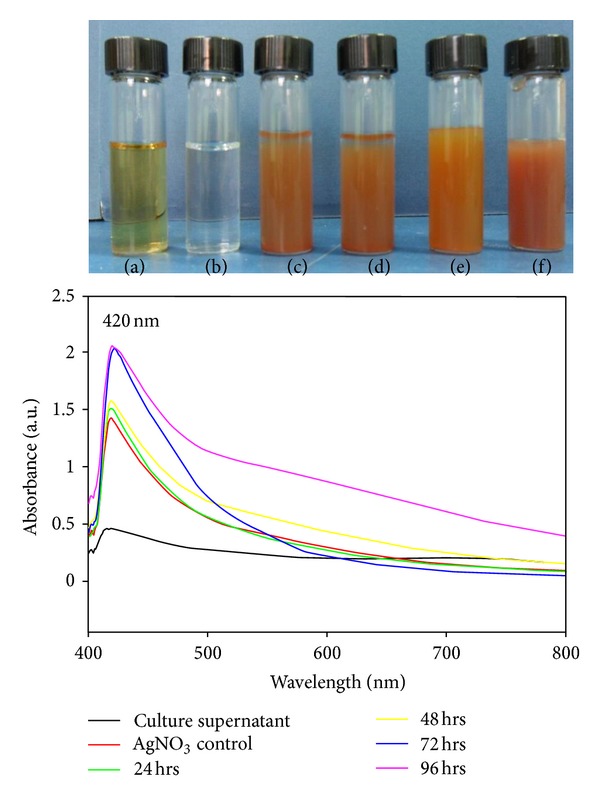
UV-Vis spectra of AgNPs synthesized using cell free supernatant of *Nocardiopsis* sp. MBRC-1. (a) Culture supernatant; (b) AgNO_3_ control; ((c)–(f)) correspond to the AgNO_3_ treated with culture supernatant incubated for 24, 48, 72, and 96 hrs, respectively.

In this study, AgNPs were successfully synthesized in the culture supernatant of *Nocardiopsis* sp. MBRC-1. Interestingly, the culture supernatant incubated with the silver nitrate mediated the biosynthesizing of AgNPs within 24 hrs of incubation. During the experiment, the pH of the sample was adjusted to 7.0. The appearance of a yellowish brown color in the silver nitrate treated flask indicated the formation of silver nanoparticles, whereas no color change was observed in either the culture supernatant without silver nitrate or the silver nitrate control experiments. Notably, the intensity of the brown color increased dramatically up to 24 hrs and was maintained throughout the experiment. This may have been due to the excitation of surface plasmon resonance (SPR) and the reduction of AgNO_3_. In the UV-visible spectrum, a strong and broad peak was observed between 420 nm, indicating the presence of AgNPs. This may have occurred due to the reduction of metal ions by secondary metabolites present in the cells. The 24, 48, 72, and 96 hrs peaks indicate the absorption spectra of biosynthesized AgNPs at different incubation times (Figure 2). Numerous reports have discussed the biosynthesis of silver nanoparticles [3, 31, 34], but to the best of knowledge, this was the first report on biosynthesis of silver nanoparticles using a novel *Nocardiopsis* sp. MBRC-1.

### 3.3. FTIR Analysis of AgNPs

FTIR spectrum analysis of AgNPs showed intense absorption bands at 3440, 2923, 2853, 1655, 1460, and 685 cm^−1^. The intense broad absorbance at 3440 cm^−1^ (O–H stretch) is the characteristic of the H-bonded functional group in alcohols and phenolic compounds. The band at 2923 and 2853 cm^−1^ (C–H stretch) can be assigned to the alkanes group. The intense medium absorbance at 1655 cm^−1^ (–C=C– stretch) is the characteristic of the alkenes group. The intense medium absorbance at 1460 cm^−1^ (C–H bend) is the characteristic of the alkanes group. The intense broad absorbance at 685 cm^−1^ (–C=C–H: C–H bend) is the characteristic of the alkynes group. A previous report reveals that the alcohols, phenolic, alkynes, and alkanes groups have a strong ability to interact with nanoparticles [31, 35, 36].

### 3.4. XRD Analysis of AgNPs

**Figure 3:**
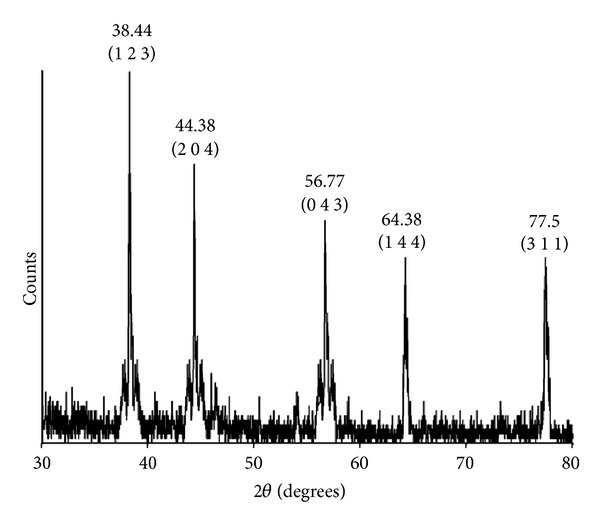
X-ray diffraction pattern of the AgNPs obtained from *Nocardiopsis* sp. MBRC-1.

The XRD pattern of the silver nitrate-treated sample (Figure 3) corresponds to that of silver nanoparticles. The XRD pattern shows five intense peaks in the whole spectrum of 2*θ* values ranging from 30 to 80. It is important to know the exact nature of the silver particles formed and this can be deduced from the XRD spectrum of the sample. XRD spectra of pure nanoparticles silver structures and pure silver nitrate have been published by the Joint Committee on Powder Diffraction Standards (file no. 04-0783). A comparison of our XRD spectrum with the standard confirmed that the silver particles formed in our experiments were in the form of nanoparticles, as evidenced by the peaks at 2*θ* values of 38.44°, 44.38°, 56.77°, 64.38°, and 77.50°, corresponding to 1 2 3, 2 0 4, 0 4 3, 1 4 4, and 3 1 1 planes for silver, respectively. The full width at half maximum (FWHM) values measured for 1 2 3, 2 0 4, 0 4 3, 1 4 4, and 3 1 1 planes of reflection was used with the Debye-Scherrer equation to calculate the size of the nanoparticles. The particle sizes obtained from XRD line broadening agreed well with those obtained from SEM. From these, the average particle size was found to be around 45 ± 0.05 nm.

### 3.5. FE-SEM Analysis of AgNPs

**Figure 4:**
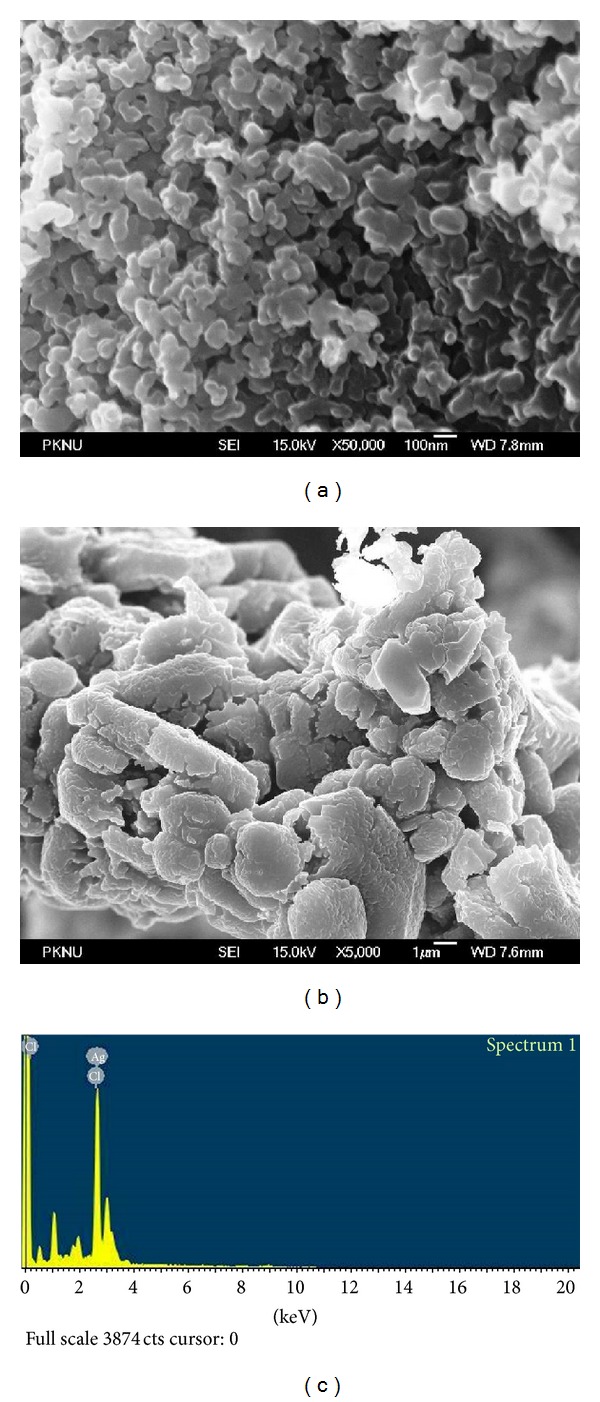
((a) and (b)) FE-SEM images of AgNPs synthesized by *Nocardiopsis* sp. MBRC-1. (a) 100 nm scale, (b) 1 *µ*m scale, and (c) EDX analysis of AgNPs synthesized by *Nocardiopsis* sp. MBRC-1.

FE-SEM determinations of the above-mentioned sample showed the formation of nanoparticles, which were confirmed to be of silver by EDX. As shown in Figures 4(a) and 4(b), well-dispersed nanoparticles could be seen in the samples treated with silver nitrate. EDX analysis also showed a peak in the silver region, confirming the formation of silver nanoparticles (Figure 4(c)). The optical absorption peak is observed approximately at 3 keV, which is typical for the absorption of metallic silver nanoparticles due to surface Plasmon resonance [37]. In addition, other peaks for Cl and O were observed which are possibly due to emissions from proteins or enzymes present in the culture supernatant [30].

### 3.6. TEM Analysis of AgNPs

**Figure 5:**
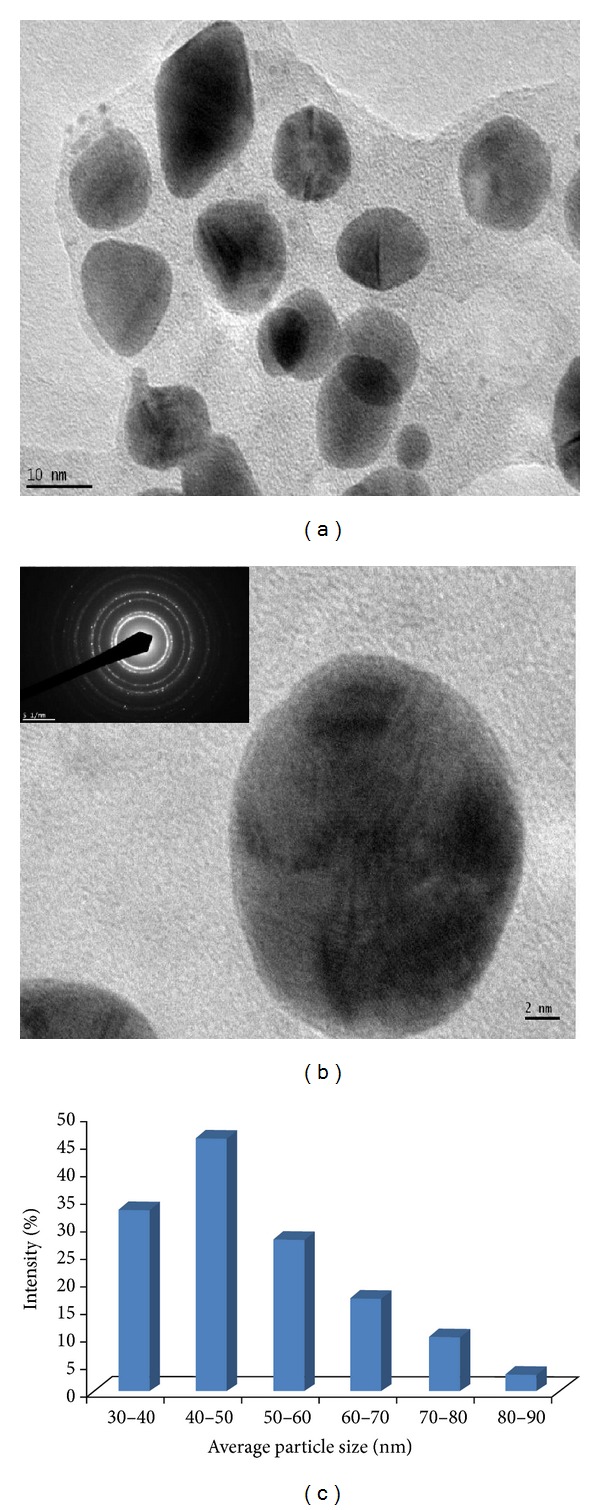
HR-TEM images of AgNPs formed by *Nocardiopsis* sp. MBRC-1. (a) 10 nm scale, (b) 2 nm scale and selected area diffraction pattern. (c) Particle-size distribution under unoptimized conditions. The particle-size distribution revealed that the particles ranging from 30 to 90 nm had the maximum intensity, and thereafter the intensity was reduced. The average particle size was found to be 45 ± 0.15 nm.

The TEM image analysis (Figures 5(a) and 5(b)) revealed that silver nanoparticles were spherical in shape. The micrograph showed NPs with variable shape; most of them present in spherical in nature. The TEM micrograph also confirmed the size of NPs, which were in the range of 30–90 nm with an average particle size of 45 ± 0.15 nm. Majority of the AgNPs were aggregates with only a few of them showing scattering of varying sizes as observed under TEM. The particle size distribution histogram plot constructed from the TEM micrograph is shown in Figure 5(c). Synthesis of AgNPs by treating AgNO_3_ solution with the culture supernatant of *K. pneumonia *(belonging to the family Enterobacteriaceae) has also been reported, in which the particles range in size from 28.2 to 122 nm and possess an average size of 52.5 nm [14]. A study on synthesis of AgNPs using *Morganella* sp. (belonging to the family *Enterobacteriaceae*) reported spherical nanoparticles of ~20 nm size [38].

### 3.7. Antimicrobial Activity of the AgNPs

In this study, the antimicrobial activity of AgNPs using a novel biosynthetic method was evaluated. In this analysis, the AgNPs displayed antimicrobial activity against a range of different pathogenic microorganisms (Table 1). The mean of three replicates of the diameter of the zone of inhibition (30 *μ*g/mL) for each microorganism was determined to be about 18.8 ± 0.30, 22.5 ± 0.10, 17.2 ± 0.15, 19.4 ± 0.20, 15.4 ± 0.15, 19.1 ± 0.20, 17.3 ± 0.25, 14.6 ± 0.20, 19.3 ± 0.20, and 22.4 ± 0.25 mm, respectively, for *Escherichia coli, Bacillus subtilis*,* Enterococcus hirae*,* Pseudomonas aeruginosa*,* Shigella flexneri*,* Staphylococcus aureus*,* Aspergillus niger*,* A. brasiliensis*,* A. fumigates*, and* Candida albicans*. The highest antimicrobial activity was observed against *Bacillus subtilis*,* Pseudomonas aeruginosa*, and* Candida albicans*. These findings are in agreement with previous studies that examined the antimicrobial activity of AgNPs against *Bacillus subtilis *and *Candida albicans* [3]. The antimicrobial activity of silver nanoparticles was reported to be due to the penetration into the bacteria, damage of cell membrane, and release of cell contents [39]. Another possibility suggested that [40, 41] was the release of silver ions from the nanoparticles, which may contribute to the bactericidal properties of silver nanoparticles.

### 3.8. Determination of Minimum Inhibitory Concentration

Minimum inhibitory concentration of AgNPs (Table 2) was evaluated against various pathogenic bacteria and fungi. The silver nanoparticles exhibited lowest minimum inhibitory concentration (MIC) against *Bacillus subtilis *at 7 *μ*g/mL, *Bacillus subtilis* 10 *μ*g/mL, and *Candida albicans* at 10 *μ*g/mL, suggesting the broad spectrum nature of their minimum inhibitory concentration. Kumar and Mamidyala [35] reported the minimum inhibitory concentration of AgNPs against Gram-positive, Gram-negative, and different *Candida* species at concentrations ranging between 4 and 32 *μ*g/mL.

### 3.9. Cytotoxic Activity

**Figure 6:**
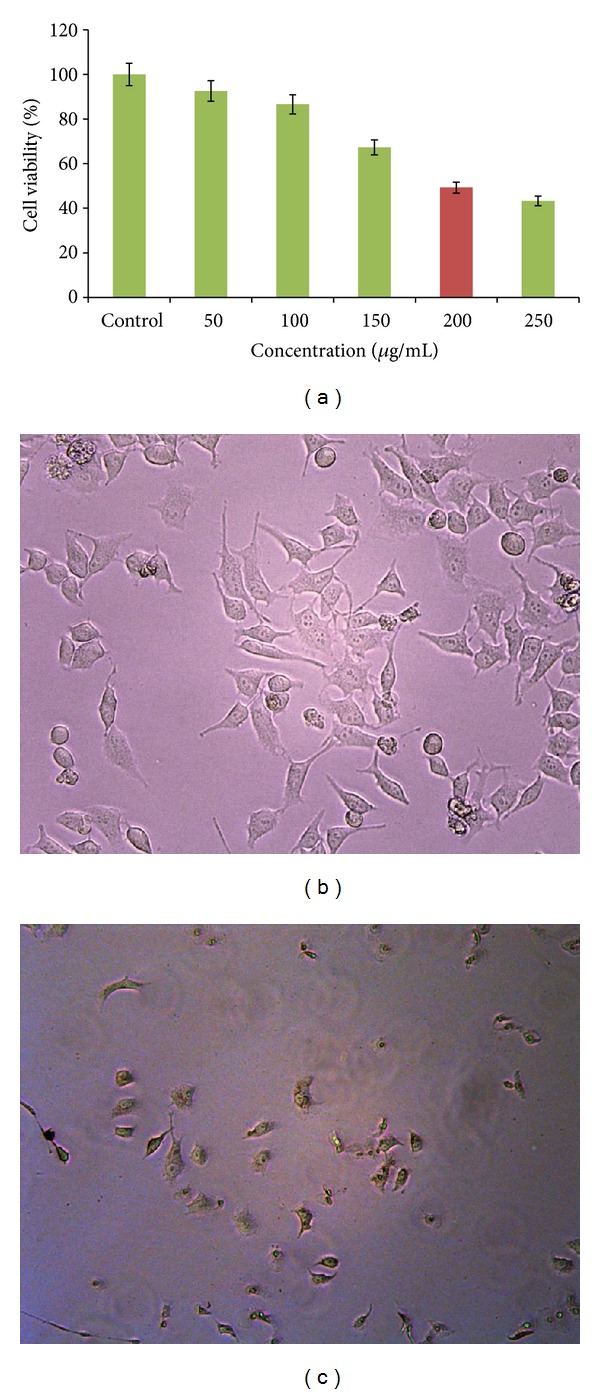
(a) MTT assay results confirming the *in vitro* cytotoxicity of AgNPs against HeLa cell lines. ((b) and (c)) Morphology of control and AgNPs treated HeLa cell lines (10x magnification). (b) Control. (c) IC_50_ concentration (200 *µ*g/mL).

The *in vitro* potential cytotoxic activity of AgNPs against cervical cancer cell lines HeLa. The use of synthetic AgNPs, there are only a few studies to determine that the cytotoxic effects of biologically synthesized AgNPs. MTT assay was used to assess the effect of AgNPs on the cytotoxicity of cancer cells. This study to evaluate the marine sediment samples isolated species *Nocardiopsis* sp. MBRC-1 derived AgNPs cytotoxicity against HeLa cancer cell lines. AgNPs inhibit the viability of the HeLa cancer cell lines in dose dependent manner. The IC_50_ value of biosynthesized AgNPs against HeLa cells at 200 *μ*g/mL concentrations (Figure 6(a)). Previously, synthesized AgNPs inducing cytotoxicity were discussed by Sriram et al. [42] and Safaepour et al. [43].

### 3.10. Cytomorphological Changes of HeLa Cells Induced by AgNPs

The morphological examinations of the HeLa cancer cells were observed and photographed using phase contrast microscope. The morphological alteration was observed in control and AgNPs treated HeLa cancer cells. The HeLa cells were treated with AgNPs at 100 and 200 *μ*g/mL concentrations for 24 hrs showing that significant morphological changes, which are characteristic features of apoptotic cells, such as loss of membrane integrity, cell shrinkage, and reduced cell density (Figures 6(b) and 6(c)).

## 4. Conclusions

In conclusion, silver nanoparticles are synthesized by the biomass of the marine actinobacterium, *Nocardiopsis* sp. MBRC-1. Marine actinobacteria are easy to handle and can be manipulated genetically without much difficulty. Considering these advantages, a bacterial system could prove to be an excellent alternative for synthesis of AgNPs. *Nocardiopsis* sp. MBRC-1 can be a good candidate for the synthesis of the AgNPs using silver nitrate of average size 45 ± 0.15 nm. *Nocardiopsis* sp. MBRC-1 genetics and enzymatic activities, sophisticated molecular breeding can produce strains and biotechnological processes, which could eliminate many types of contaminants in an economical, efficient, and simple process and environmentally friendly manner. The biosynthesized silver nanoparticles showed excellent antimicrobial activity and possessed considerable cytotoxic effect against *in vitro* HeLa cancer cell lines. IC_50_ value was found to be 200 *μ*g/mL of AgNPs against HeLa cell lines. The data represented in our study contribute to a novel and unexplored area of nanomaterials as alternative medicine. Furthermore, the biosynthesized AgNPs displayed a pronounced antimicrobial and cytotoxicity activity against clinical pathogenic microorganisms and HeLa cancer cell lines. Taken together, the data collected in this study suggests that it would be important to understand the mode of action of the biosynthesized nanoparticles prior to their use in nanomedicine applications.

## Supplementary Material

Supplemental Figure 1. Biosynthesis, characterization and biomedical applications of silver nanoparticles by marine actinobacterium *Nocardiopsis* sp. MBRC-1.

## Figures and Tables

**Table 1:** Antimicrobial activity of the AgNPs against various pathogenic micro-organisms. The data is presented as the mean ± value standard deviation of three replicates.

Micro-organisms	Zone of inhibition (mm in diameter)					
	10 *µ*g/mL	20 *µ*g/mL	30 *µ*g/mL	40 *µ*g/mL	50 *µ*g/mL	Antibiotics 30 *µ*g/mL
Bacteria						Amoxicillin
*Escherichia coli* ATCC 10536	7.5 ± 0.35	15.2 ± 0.31	18.8 ± 0.30	23.3 ± 0.20	27.3 ± 0.15	19.3 ± 0.10
*Bacillus subtilis* ATCC 6633	11.2 ± 0.35	19.4 ± 0.25	22.5 ± 0.10	28.1 ± 0.20	33.2 ± 0.20	23.8 ± 0.25
*Enterococcus hirae* ATCC 10541	6.3 ± 0.20	13.3 ± 0.14	17.2 ± 0.15	21.8 ± 0.30	25.4 ± 0.25	19.5 ± 0.10
*Pseudomonas aeruginosa* ATCC 27853	9.1 ± 0.15	17.7 ± 0.30	19.4 ± 0.20	23.6 ± 0.35	28.3 ± 0.20	21.3 ± 0.30
*Shigella flexneri* ATCC 12022	5.2 ± 0.20	11.2 ± 0.21	15.4 ± 0.15	19.3 ± 0.25	22.5 ± 0.10	17.5 ± 0.30
*Staphylococcus aureus* ATCC 6538	7.8 ± 0.25	15.1 ± 0.32	19.1 ± 0.20	24.2 ± 0.20	27.1 ± 0.15	21.3 ± 0.10

Fungi						Nystatin
*Aspergillus niger* ATCC 1015	6.7 ± 0.32	13.6 ± 0.22	17.3 ± 0.25	21.4 ± 0.20	25.3 ± 0.15	18.1 ± 0.10
*A. brasiliensis* ATCC 16404	4.8 ± 0.25	10.2 ± 0.15	14.6 ± 0.20	19.4 ± 0.10	23.4 ± 0.15	15.8 ± 0.30
*A. fumigates* ATCC 1022	7.2 ± 0.35	15.4 ± 0.22	19.3 ± 0.20	24.3 ± 0.10	26.3 ± 0.30	21.4 ± 0.15
*Candida albicans *ATCC 10231	9.5 ± 0.20	18.1 ± 0.21	22.4 ± 0.25	25.2 ± 0.25	28.4 ± 0.25	24.5 ± 0.20

**Table 2:** Minimum inhibitory concentration of the AgNPs against various bacterial and fungal strains. The data is presented as the mean ± value standard deviation of three replicates.

Micro-organisms	Minimum inhibitory concentration	
	AgNPs (*µ*g/mL)	Antibiotics (*µ*g/mL)
Bacteria		Amoxicillin
* Escherichia coli *ATCC 10536	13	11
* Bacillus subtilis *ATCC 6633	7	6
* Enterococcus hirae *ATCC 10541	16	14
* Pseudomonas aeruginosa *ATCC 27853	10	9
* Shigella flexneri *ATCC 12022	18	15
* Staphylococcus aureus *ATCC 6538	14	12

Fungi		Nystatin
* Aspergillus niger *ATCC 1015	16	14
* A. brasiliensis *ATCC 16404	18	16
* A. fumigates *ATCC 1022	13	12
* Candida albicans *ATCC 10231	10	7
