# Supplementary figures and images for: Biosynthesis, Antimicrobial and Cytotoxic Effect of Silver Nanoparticles Using a Novel Nocardiopsis sp. MBRC-1
Source: Biomed Res Int. 2013 Jul 11;2013:287638. doi: 10.1155/2013/287638 (PMC3727093; doi:10.1155/2013/287638)

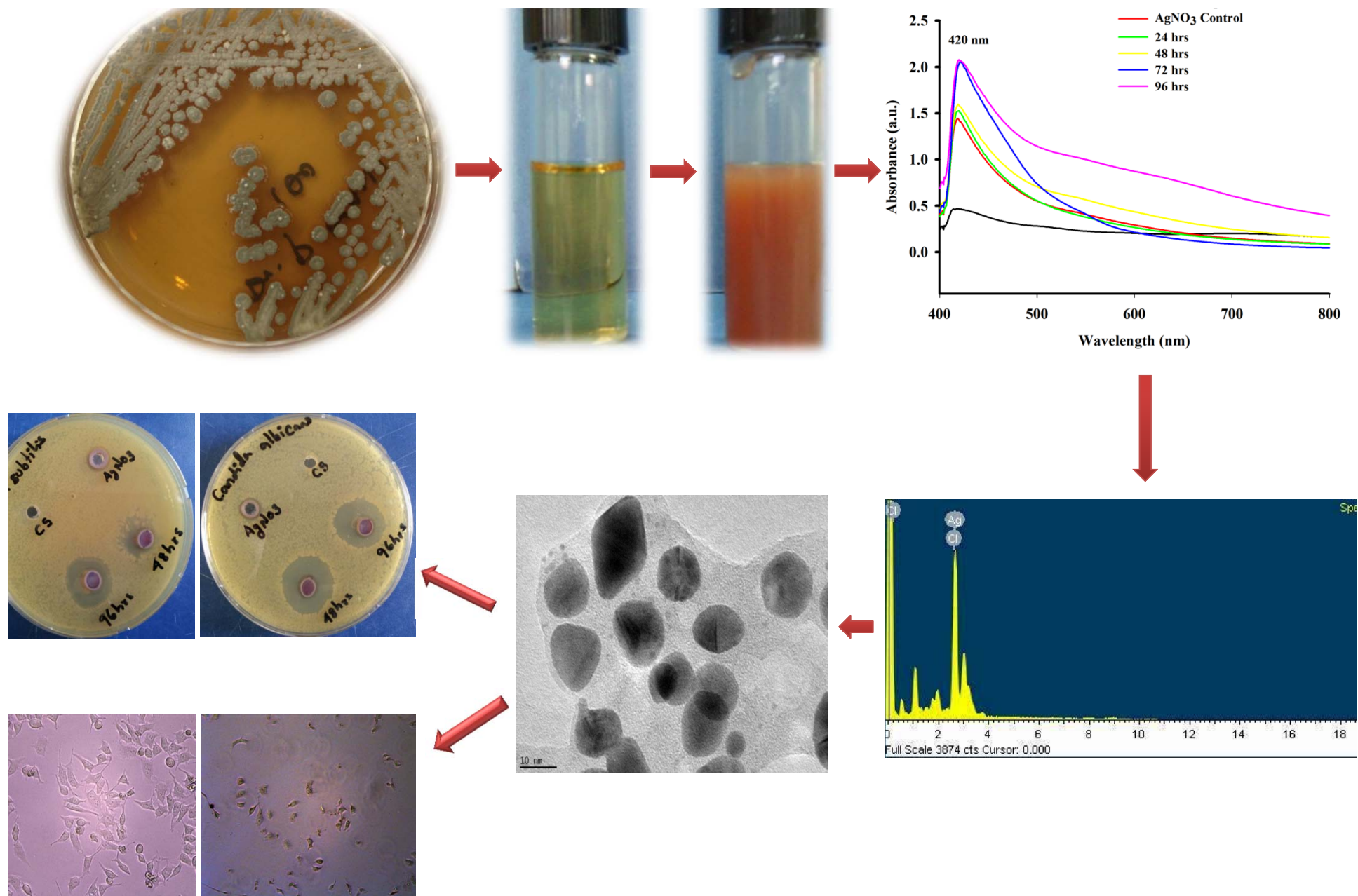

**Supplemental Figure 1. Biosynthesis, characterization and biomedical applications of silver**

Supplement: Supplementary file 1 — Supplemental Figure 1. Biosynthesis, characterization and biomedical applications of silver nanoparticles by marine actinobacterium Nocardiopsis sp. MBRC-1. [file 287638.f1.pdf]
